# Cryo-EM and AFM visualize linear polyorganophosphazene: individual chains and single-chain assemblies with proteins

**DOI:** 10.21203/rs.3.rs-3411603/v1

**Published:** 2023-10-31

**Authors:** Alexander Andrianov, Raman Hlushko, Edvin Pozharski, Vivek Prabhu

**Affiliations:** University of Maryland; University of Maryland; University of Maryland School of Medicine; National Institute of Standards and Technology

## Abstract

Polyorganophosphazenes are biodegradable macromolecules with potent immunoadjuvant activity that self-assemble with protein antigens to provide biological activity. Direct imaging by cryogenic electron microscopy reveals the coil structure of the highly-charged high molecular mass synthetic polyorganophosphazenes within the vitrified state without any additives for contrast enhancement for the first time. Upon mixing with protein antigens under a controlled stoichiometric ratio, multiple proteins bind at the single chain level revealing a structural change reminiscent of compact spherical complexes or stiffened coils depending on the bound protein antigen. The structural outcome depends on the protein charge density that cannot be deduced by methods, such as dynamic light scattering, thus revealing direct morphological insight necessary to understand *in vivo* biological activity. Complementary atomic force microscopy supports the binding morphology outcomes as well as additional analytical techniques that indicate binding. These observations open opportunities to understand supramolecular assembly of proteins and other biomacromolecules at the single chain level with highly charged polyelectrolytes for vaccines as well as important to developing fields such as polyelectrolyte complex coacervation.

## Introduction

Polymer architecture and conformation - the spatial arrangement of the atomic groups in an individual chain - constitute the fundamental molecular basis, which essentially defines or significantly impacts physical and biological behavior of synthetic macromolecules^1, 2, 3, 4, 5^. Although the conformational analysis and direct visualization of naturally assembled rigid and semirigid macromolecular structures, such as proteins and polynucleotides, has been rapidly evolving, flexible synthetic polymers remain somewhat outside of this success story^6^. The selection of tools for studying, visualizing and controlling conformation of single-chain molecules of flexible synthetic polymers in solutions is still severely limited, mainly due to the propensity of these macromolecules to fold and aggregate under the conditions of the analysis^6^. Nevertheless, structural and conformationally dynamic features of flexible chains, as they are exposed to various types of application related activities, can provide important clues to optimizing their performance and understanding their mechanism of action, which is especially important for physiologically active macromolecules and their interactions with living systems.

Poly[di(carboxylatophenoxy)phosphazene], PCPP is a synthetic ionic macromolecule, which stands out from conventional polyelectrolytes due to its potent immunoadjuvant activity, which has been demonstrated in numerous *in vivo* studies and in the clinical environment^7, 8, 9^. The accumulated physico-chemical, biophysical and *in vivo* data reveal that PCPP realizes its biological activity through spontaneous self-assembly with antigenic proteins in aqueous solutions^7^. However, the comprehensive structural characterization of PCPP in aqueous solutions and its supramolecular assemblies with proteins has not yet been carried out. It is expected that understanding the mechanism of supramolecular assembly and detailed characterization of the resulting structures remains one of the key prerequisites for the successful development and optimization of this vaccine delivery system. Furthermore, insights on conformational changes in the system can broaden our knowledge of intermolecular interactions between proteins and polyelectrolytes in general and play an important role in modulating biological performance of polyelectrolyte-based biomaterials.

Cryogenic Electron Microscopy (cryo-EM) and Atomic Force Microscopy (AFM) remain some of the most advanced and prevalent techniques for visualizing single biomacromolecules. Cryo-EM provides real-space microscopy images of biomolecules embedded in vitreous, glass-like ice shedding light on the behavior of macromolecules in their native environment^10, 11, 12, 13^. The technique is rapidly becoming an attractive method in the field of structural biology with molecular structures as small as the 52 kDa streptavidin and the 40 kDa ribonucleic acid (RNA) are within the practical limits of characterization^10^. More recently, cryo-EM has also become a game-changing tool not only in protein structure determination, but also in protein assemblies, protein ligand–receptor interactions, and, now, assemblies of synthetic peptides.^14, 15^ However, being developed as a predominantly biological technique, the use of cryo-EM to study synthetic polymers and soft matter has been much more limited mainly due to insufficient contrast in such systems^16, 17^ and their inherent flexibility that prevents robust multi-particle averaging that is key to atomic resolution single particle reconstruction of protein molecules. Most of cryo-EM research on synthetic polymers has been focused on investigations of high mass contrast systems, such as helical^17^ and dendronized^18, 19, 20, 21^ polymers, spherical^22^ or cylindrical^23, 24, 25^ polyelectrolyte brushes with dense side chains, various micellar assemblies^26, 27, 28, 29^ and aggregates formed by polyelectrolyte complexes^30^ or stacking of the conjugated polymer in organic solvents^31^. In contrast, AFM has been successfully employed for imaging single-chain linear synthetic polyelectrolytes, however this technique only allows visualization of macromolecules in their adsorbed state^32, 33, 34, 35^. It can be expected that the complementary information provided by two methods, which exploit distinctly different experimental techniques, can offer invaluable insights into solution behavior of PCPP and its self-assembly with model protein systems. Alternate approaches, such as small-angle neutron scattering provide average chain conformation and rely on isotopic labeling in order to directly measure the form factor of a chain molecule, which is related by Fourier transform to the pair distribution function and by convolution to real-space density distribution^36^. Therefore, models must be applied to relate the reciprocal space to the real-space trial structure due to the classical phase problem in scattering.

Here we successfully visualized and compared images of individual chains of a linear synthetic polymer, PCPP, in aqueous solution in its vitrified and surface adsorbed states. Both cryo-EM and AFM methods demonstrated spontaneous association of PCPP with model antigenic proteins - bovine serum albumin (BSA) and hen egg lysozyme. Two distinct scenarios of complex formation were revealed. While self-assembly with BSA occurred without noticeable changes in polymer conformation, significant compaction of linear chains was observed for lysozyme. This was manifested in the formation of loops and re-crossed chains by cryo-EM and formation of anisotropic hemispheres of adsorbed complexes by AFM. Given previously demonstrated *in vivo* immunopotentiating effect of PCPP with either of the studied proteins^37, 38^, the structural insights provided by cryo-EM and AFM can offer important clues in studying the mechanism of biological activity of this important macromolecular immunoadjuvant. Such measurements offer unique data needed to interpret measurements, such as dynamic light scattering and small-angle X-ray and neutron scattering in polyelectrolyte-protein complex solutions.

## Results

### Direct visualization of individual PCPP chains using cryo-EM and AFM methods.

**Figure 1:**
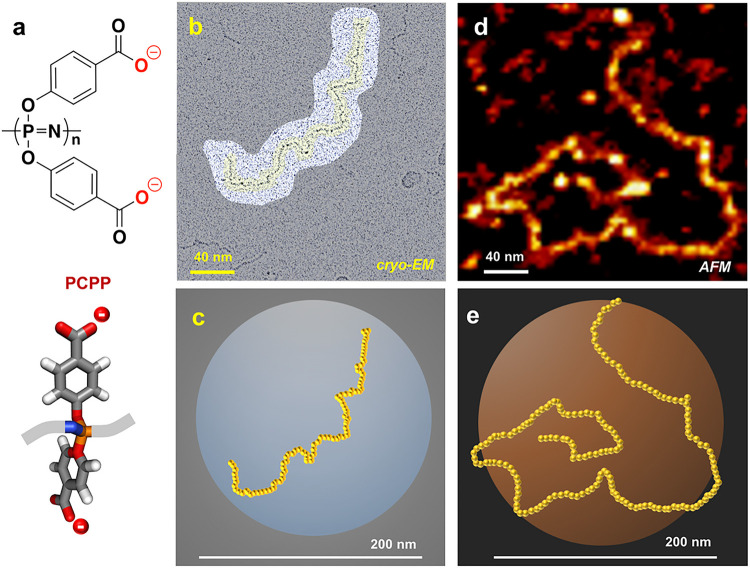
Visualization of individual PCPP chains. (a) Chemical structure of PCPP repeat unit. (**b**) Cryo-EM image of a single PCPP chain and (**c**) its artistic reproduction. (**d**) AFM image of a single PCPP chain and (**e**) its artistic reproduction.

PCPP is a weak synthetic polyelectrolyte with the mass-average molar mass of 800,000 g/mol, which is fully soluble under neutral and basic conditions^39^. Its molecular structure includes two carboxylate moieties per repeat unit (Fig. 1a). Cryo-EM images of PCPP samples prepared in a vitrified state from solution in a phosphate buffer at pH 7.4 show clearly visible polymer chains (Fig. 1a–b and Supplementary information, Fig. S1). Their appearance can be described as random coils of various length with the majority of them exceeding 100 nm in size. Some loose loops can be noticed in the images, but no apparent re-crossing is visible.

Investigation of PCPP chains by AFM was initially impeded by difficulties in adsorbing the anionic polyphosphazene macromolecule on the surface of freshly-cleaved mica. To obtain sufficient adhesion of PCPP to the inorganic surface, mica was modified with bovine serum albumin (BSA) (Supplementary information, Fig. S2). AFM images of PCPP adsorbed on such surfaces are shown in Fig. 1d–e and in Supplementary information, Fig. S3. Although, the dimensions of adsorbed PCPP chains appear to be somewhat larger than those detected in vitrified samples, PCPP coils visualized by both methods appear to be of similar shape. This may reflect that adsorption of PCPP is favored for higher molecular weights with the smaller chains rinsed off during the sample preparation step.

### Self-assembly of PCPP-BSA complexes as revealed by cryo-EM.

**Figure 2:**
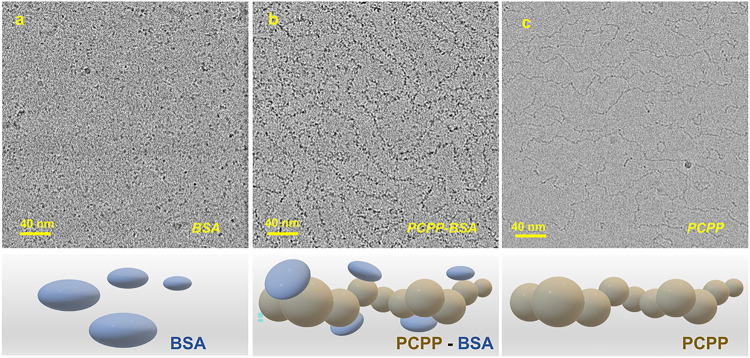
Contrasting of PCPP with BSA. Cryo-EM images of (a) BSA, (b) PCPP-BSA and (c) PCPP (c) (2.5 mg/mL PCPP, 0.25 mg/mL BSA, 50 mmol phosphate buffer, pH 7.4).

To evaluate the potential effect of BSA on PCPP chains visualized by AFM, protein-polymer solutions in their vitrified form were also studied by cryo-EM. BSA, a globular protein with molecular mass of 66 kDa, is visible in cryo-EM images as small dots randomly scattered over the field (Fig. 2a). Previously BSA was characterized with small-angle neutron scattering as a prolate ellipsoid with *a* = (7.0 ± 0.2) nm and *b* = (2.0 ± 0.1) nm with the radius of gyration of 3.05 nm, where *a* and *b* are the semi-major and minor axes, respectively.^40^ The addition of PCPP to BSA solution results in the reorganization of such dots via their structuring along curved lines (Fig. 2b). These densely dotted lines resemble chains of free PCPP visualized under the same experimental conditions (Fig. 2c), but show a superior contrast. Essentially, the approach can be considered as ‘staining’ of PCPP chains through the association of synthetic macromolecule with BSA. These results are in line with previous reports on noncovalent association of BSA and PCPP demonstrated by size exclusion chromatography and asymmetric flow field flow fractionation (AF4)^37, 41^. The comparison of Fig. 2b and Fig. 2c suggests no significant ‘disturbing’ effect of BSA binding on the conformation of the polymer. These results also indicate that any potential influence of BSA on the conformation of PCPP chains shown in AFM generated images (Fig. 1d and Supplementary information, Fig. S3) is minor.

### Chain statistics.

To quantify the flexibility of PCPP the imaged polymeric chains were traced to provide backbone contours for analysis of conformation. The tracing and conformational analysis was performed according to SmarTrace algorithm.^42^ Here the traced chains were randomly segmented to nonoverlapped fragments of various contour length. Then for all of the segments squared end-to-end distance < *R*^2^*(s)* > and the tangent vector correlation < cosθ*(s)* > length-dependent trends were compared with the predictions of the standard worm-like chain (WLC) model for polymers (Supplementary information, Fig. S4-S6). The persistence lengths for PCPP and PCPP/BSA are presented in Table 1.

### Interactions of PCPP with positively charged protein - lysozyme.

**Figure 3:**
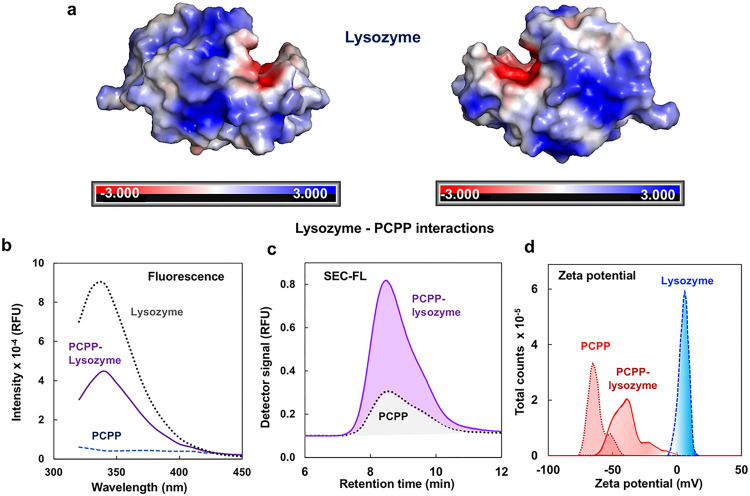
Interactions of PCPP with lysozyme. (**a**) Schematic presentations of electrostatic potential surfaces of hen egg lysozyme^45^ (images generated using PyMOL 2.5.5 (Schrödinger Inc., New York, NY); (**b**) Fluorescence of lysozyme - dotted line, PCPP-lysozyme complex (1:7 mole/mole) - solid line and PCPP - dashed line (0.5 mg/mL PCPP, 0.06 mg/mL lysozyme, 50 mM phosphate buffer, pH 7.4, excitation wavelength: 280 nm); (**c**) size-exclusion chromatography profiles of PCPP-lysozyme - solid line and PCPP - dotted line(0.5 mg/mL PCPP, 0.06 mg/mL lysozyme, 50 mM phosphate buffer, pH 7.4) (**d**) zeta-potential distribution profiles for PCPP - dashed line, lysozyme - dotted line and their mixture -solid line (0.5 mg/mL PCPP, 1 mg/mL lysozyme, complex: 0.5 mg/mL PCPP and 0.06 mg/mL lysozyme, 50 mM phosphate buffer, pH 7.4)

Hen egg lysozyme, a protein with a large positively charged surface area (Fig. 3a), has been shown to form complexes with PCPP at neutral pH^43,44^. Although such interactions typically result in a severe aggregation and phase separation^43^, PCPP-lysozyme mixtures prepared at 1:7 polymer-to-protein mole ratio are essentially clear solutions with no aggregates detected by dynamic light scattering (DLS)(Fig. S7). Fluorescence quenching data and size exclusion chromatography analysis provide clear indication of binding in the system (Fig. 3b–c), while zeta potential distribution profiles suggest that this is an electrostatically driven process (Fig. 3d). Nevertheless, the above-mentioned data, along with the isothermal calorimetry results indicating the dissociation constant of the complex below the micromolar range (Supplementary information, Fig. S8 and Table S1), do not yet constitute a proof of the single-chain complex formation as the co-existence of free PCPP chains and larger aggregates still cannot be ruled out completely. To that end, the analysis of complexes using cryo-EM and AFM methods could provide critical information on the mechanism of complexation in this system, which is important for further understanding of the mechanism of immunoadjuvant activity of PCPP.

**Figure 4:**
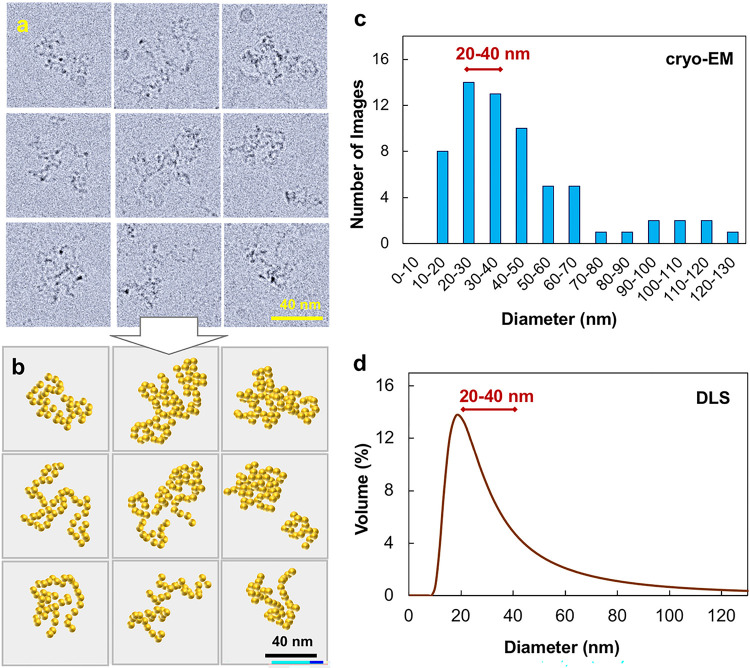
Visualization and characterization of PCPP complexes with lysozyme. (**a-b**) Cryo-EM images of PCPP-lysozyme complexes (1:7 mole ratio, 0.5 mg/mL PCPP and 0.06 mg/mL lysozyme) and their artistic reproduction. (**c-d**) complex size distribution as determined by measuring pervaded diameters of coils in cryo-EM images and DLS.

Representative cryo-EM images of vitrified samples of PCPP-lysozyme mixtures (1:7 mole ratio) and their artistic reproductions (skeletal main chain trajectory) are shown in Fig. 4a–b. The absolute majority of coils visible in the field are compact coils with dimensions significantly smaller than for free PCPP chains described above. The conformations show extensive looping and apparent re-crossing of chains. The size distribution obtained by encircling the images of individual coils (pervaded volume), (as shown in Supplementary information, Fig. S9) indicate that the majority of them can be characterized with diameters in the range of 20 nm to 40 nm (Fig. 4c). The size distribution obtained from cryo-EM measurements was compared with the results of DLS studies, in which the volume distribution was crudely assessed using Mie theory assuming spherical shape and homogeneity of complexes^46^ (Fig. 4c). The comparison of results shows close similarity between distribution profiles, although DLS assessment showed that the majority of complexes were in 10 nm to 30 nm diameter range.

**Figure 5:**
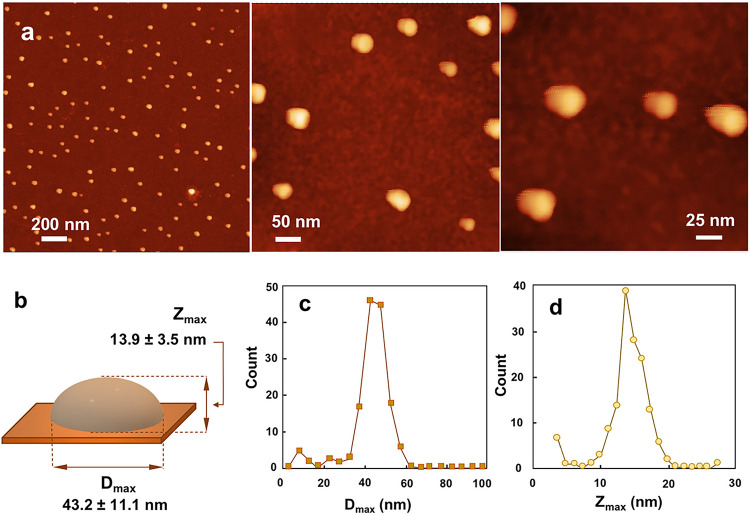
Visualization and characterization of PCPP using AFM. (**a**) AFM images at various magnifications. (**b**) Schematic presentation of adsorbed complex with average dimensions. (**c-d**) Diameter (solid-colored squares) and (open circles) height distribution of complexes (AFM images are taken from bPEI-treated mica substrates).

AFM visualization of PCPP-lysozyme complexes was initially attempted using BSA-mediated adsorption on mica surface - the technique described above for free PCPP chains. Although some AFM images of complexes were collected (Supplementary information, Fig. S10), the best adsorption and image resolution was obtained by using mica surface pretreated with branched polyethyleneimine (bPEI) (Fig. 5a). In either case, the surface adsorbed images do not reveal individual polymer chains, but rather showed hemispheres with an average diameter of around 40 nm and 14 nm average height (Fig. 5b–d). Although, AFM data provide somewhat more limited information compared to cryo-EM images, the results show a compaction of PCPP chains upon addition of lysozyme. The dimensions of the complexes as visualized by cryo-EM and AFM are comparable, especially if the anisotropy of the hemisphere (larger pervaded diameter) is attributed to the effect of surface adsorption in the sample preparation process. It should also be noted that similarly to protein molecules, PCPP could partially adsorb at the water-air interface ^47, 48, 49^ resulting in some increase of its apparent size in cryo-EM images.

## Discussion

The evolving interest in PCPP and its structural analogs is primarily dictated by the potent immunoadjuvant effect demonstrated by this biodegradable macromolecule in multiple animal models and in clinical trials^7, 8, 9^. Immunoadjuvants are important components of contemporary vaccines, which enhance, prolong and modulate antigen-specific immune responses^50, 51, 52^. Although, the exact mechanisms of action still remain under discussion, the antigen delivery capabilities constitute some of the key features for many representatives of the class^53^. In particular, dispersed systems, such as aluminium hydroxide or phosphate (Alum or Alhydrogel), nanoemulsions, liposomes, polymer nanoparticulate are known to be capable of effective presentation of the antigen. Their dimensions and well-defined surface, on which the antigen is displayed, provide for virus-mimicking characteristics and largely enabling uptake by immunocompetent cells^53^. PCPP - a linear flexible macromolecule appears to lack those essential features, but nevertheless outperformed many of particulate adjuvants *in vivo*. Visualization of macromolecular architecture and conformation of PCPP, as well as their complexes with proteins in solutions constitute important prerequisites for understanding the mechanism of activity of this class of adjuvants.

Cryo-EM of conventional synthetic polymers, except of some highly branched structures and aggregates, has not been sufficiently advanced due to low mass contrast exhibited by most conventional macromolecules with carbon-carbon backbones. To that end, PCPP offered some interesting possibilities since it displays some structural similarities to DNA, which shows high mass contrast in cryo-EM studies due to its phosphorus containing backbone and electron-dense base pairs^20^. In fact, the phosphorus-nitrogen backbone of PCPP along with two aromatic side groups per repeat unit, enabled direct visualization of its macromolecular coils in samples prepared by vitrification of solutions at near physiological pH. To the best of our knowledge, this is the first report on a direct visualization of single chains of a linear synthetic polymer by cryo-EM. The observed images of individual chains were consistent with those obtained by AFM studies using surface adsorbed PCPP.

Further study on interactions of PCPP with BSA and lysozyme provides a direct proof of polymer association with proteins in aqueous solution. Moreover, in either case, the formation of complexes on the basis of a single polymer chain was observed with the absence of larger multi-chain aggregates. It has to be noted that both proteins selected for the present study have been previously successfully adjuvanted by PCPP in immunogenicity studies *in vivo*^37, 38^. Therefore, the results provide an important information relevant to the ongoing discussion on the mechanism of action of polyphosphazene adjuvants. The study revealed two distinct scenarios of the way PCPP spontaneously self-assembles with proteins in aqueous solution. BSA, a protein with an overall net-negative charge at physiological pH, associates with PCPP without introducing significant conformation changes in the polymer (Fig. 2). In contrast, interactions with lysozyme, which has a net-positive charge at neutral pH, are sufficiently strong to cause compaction of PCPP chains, which is demonstrated by both cryo-EM and AFM visualization (Figs. 4 and 5). Regardless of the resulting complex conformation, PCPP maintains its ability to enhance immunogenicity of either of these proteins^37, 38^. Direct measurements will enable informed interrelationships on polyelectrolyte-protein binding mechanisms driven by electrostatic interactions or hydrophobicity and solvent quality arguments that may occur as in this work at the single chain level versus macrophase separation^54, 55^.

## Conclusions

The determination of vaccine particle structure represents a modern characterization challenge and world-wide interest. Advances in cryo-EM have enabled characterization of biomacromolecules in the vitrified state due to sufficient electron density contrast between the compact protein globule, lipid, DNA, or RNA and surrounding water. It was demonstrated that the phosphorus and nitrogen containing main chain, intrinsic to polyorganophosphazenes, enables substantial electron microscopy contrast to directly observe single chains. The spontaneous self-assembly with antigenic proteins in the form of single-chain complexes was observed for the first time under conditions of 1:7 stoichiometry of mixing without the presence of multichain aggregates. The protein ampholytic character with charge heterogeneity through the BSA (net-negative) and lysozyme (net-positive) examples had contrasting single chain complex structures that offers a more detailed view of the binding characteristics and role of polyelectrolyte charge and design. The stiffening of PCPP upon BSA binding represents a new mechanism that requires computer simulation modeling on how an already highly charged polyelectrolyte further stiffens as revealed by the persistence length estimates, whereas lysozyme leads to compact objects, reminiscent of condensation. In either case, the single chain complexes maintain immunoadjuvant potency due to the flexible and conformational dynamics that allow access to the antigenic protein.

## Methods

### Materials.

Lysozyme from chicken egg white, BioUltra, lyophilized powder, ≥98% and bovine serum albumin, lyophilized powder, ≥96% (Sigma-Aldrich, Saint Louis, MO) were used as received. High molecular weight poly[di(carboxylatophenoxy)phosphazene], PCPP (800,000 g/mol) was synthesized as described previously. ^39, 56^

### Cryogenic Electron Microscopy.

A sample droplet of 3.0 μL was deposited on holey carbon film TEM grids (Q3100CR1.3-2nm, Electron Microscopy Sciences, Hatfield, PA), which were plasma-treated beforehand using glow discharge PELCO EasiGlow (Ted Pella Inc., Redding, CA) for 15 s at 10 mA. The grids were then double blotted for 2 s on the Vitrobot (Vitrobot Mark IV, FEI, Hillsboro, OR), leaving a thin layer stretched over the grid holes and vitrified in liquid ethane. The temperature was monitored and kept constant in the chamber during all the sample preparation steps. The samples then were transferred to 200 kV Talos Arctica (FEI, Hillsboro, OR) equipped with FEI Falcon3EC direct electron detector. Imaging was performed at temperature about 90 K at an acceleration voltage of 200 kV. The data were collected using EPU software and processed in CryoSPARC 4.2.1 (Structura Biotechnology Inc., Toronto, Canada). ImageJ software^57^ was later used to tune the contrast and brightness of the acquired images.

### Atomic Force Microscopy.

AFM studies were performed using a Bruker Dimension Icon AFM instrument (Bruker, Billerica, MA) in the ScanAsyst mode. The cantilever used for measurements was a ScanAsyst Air probe (Bruker, Billerica, MA) with resonant frequency 70 kHz, spring constant 0.4 N/m, and with a nominal tip radius of 2.0 nm. The PCPP samples were deposited by dip coating from BSA/PCPP solution (BSA 0.25 mg/mL, PCPP 0.05 mg/mL) on freshly cleaved muscovite mica V1 (71856-01, Electron Microscopy Sciences, Hatfield, PA) for 5 min deposition time followed by rinsing and drying in nitrogen flow. The PCPP-lysozyme complexes were deposited by dip coating from (lysozyme 0.006 mg/mL, PCPP 0.05 mg/mL) solution on silicon wafers (⟨111⟩, 0.5 mm, Institute of Electronic Materials Technology, Poland) primed with BPEI for 5 min time followed by drying in nitrogen flow.

### Dynamic light scattering and Zeta potential.

DLS and Zeta potential measurements were performed using a Malvern Zetasizer Nano ZS (Malvern Instruments Ltd., Worcestershire, UK) using a 532 nm laser with a back-scattering detector an angle of 173° with data recorded and processed in Malvern Zetasizer Software 7.10. Polymer and protein were filtered through poly(vinylidene fluoride) (PVDF) Millex syringe filters (EMD Millipore, Billerica, MA) with a pore size of 0.22 μm prior to measurements. DLS measurements were performed at 25 °C using BRAND (BrandTech Scientific, Inc., Essex, CT) disposable ultra-micro cuvettes with plastic caps for polymer and complex formulations containing 0.5 mg/mL PCPP and a protein concentration of 0.5 mg/mL in 50 mM phosphate buffer, pH 7.4. Each measurement was averaged from 12 scans. Zeta potential measurements were performed at 25 °C using Malvern DTS1070 cuvettes (Malvern Instruments Ltd., Worcestershire, UK) for the same concentrations with the accumulation and averaging of 100 scans.

### Isothermal Titration Calorimetry.

ITC experiments were performed using a Nano ITC SV instrument (TA Instruments, Waters, New Castle, DE, USA) at 25 °C. In a typical experiment, 900 μL of PCPP solution was placed into isothermal chamber and was titrated by 10 μL aliquots of Lysozyme from a 250 μL syringe rotating at 36.7 rad/s with a 300 s delay between each injection. Each injection generated a heat release curve (microjoules per second versus seconds), which later was processed using NanoAnalyze software, version 3.12.5 (TA Instruments, Waters, New Castle, DE, USA) to yield the heat associated with each injection. Data analysis was performed with the above software and a single set of identical sites (SSIS) binding model was used to calculate thermodynamic parameters: binding constant (K_d_), reaction stoichiometry (n), enthalpy (ΔH), and entropy (ΔS).

### Fluorescence.

Fluorescence measurements were performed using a BioTek SyNergy neo2 multimode reader (BioTek Instruments, Inc., Winooski, VT). Emission spectra were recorded at a fixed excitation wavelength of 280 nm.

### Size-exclusion chromatography.

SEC analysis of polymer and polymer/protein complex were conducted using an Agilent 1260 Infinity II Binary LC system (Agilent, Santa Clara, CA) equipped with a G7112B binary pump, G7167A Multisampler, G7116A multicolumn thermostat, G7117C diode array detector, G7121A fluorescence detector, and TSKgel GMPW size-exclusion column (Tosoh Bioscience, LLC, Japan); phosphate buffer (PBS) with 10 % acetonitrile volume fraction used as a mobile phase.

## Figures and Tables

**Table 1.** Persistence lengths values (*I*_p_) of PCPP obtained on the basis of AFM and cryo-EM measurements (the uncertainty (±) in the persistence length is given by one standard deviation from the average value).

Polymer	*I*_p_ (nm)
PCPP-BSA (AFM)	17.8 ± 0.5
PCPP-BSA (Cryo-EM)	14.8 ± 0.3
PCPP (Cryo-EM)	7.7 ± 0.7
